# Co-delivery of paclitaxel (PTX) and docosahexaenoic acid (DHA) by targeting lipid nanoemulsions for cancer therapy

**DOI:** 10.1080/10717544.2021.2018523

**Published:** 2021-12-29

**Authors:** Bo Li, Tingfei Tan, Weiwei Chu, Ying Zhang, Yuanzi Ye, Shanshan Wang, Yan Qin, Jihui Tang, Xi Cao

**Affiliations:** a Department of Pharmacy, The First Affiliated Hospital of Anhui Medical University, Hefei, People’s Republic of China; b The Grade 3 Pharmaceutical Chemistry Laboratory of State Administration of Traditional Chinese Medicine, Hefei, People’s Republic of China; c Department of Pathology, The First Affiliated Hospital of Anhui Medical University, Hefei, China; d School of Pharmacy, Anhui Medical University, Hefei, China

**Keywords:** Paclitaxel (PTX), docosahexaenoic acid (DHA), folic acid (FA), lipid nanoemulsions (LNs), breast cancer, multi-drug chemotherapy

## Abstract

Breast cancer is one of the most common types of cancer in female patients with high morbidity and mortality. Multi-drug chemotherapy has significant advantages in the treatment of malignant tumors, especially in reducing drug toxicity, increasing drug sensitivity and reducing drug resistance. The objective of this research is to fabricate lipid nanoemulsions (LNs) for the co-delivery of PTX and docosahexaenoic acid (DHA) with folic acid (FA) decorating (PTX/DHA-FA-LNs), and investigate the anti-tumor activity of the PTX/DHA-FA-LNs against breast cancer both *in vitro* and *in vivo*. PTX/DHA-FA-LNs showed a steady release of PTX and DHA from the drug delivery system (DDS) without any burst effect. Furthermore, the PTX/DHA-FA-LNs exhibited a dose-dependent cytotoxicity and a higher rate of apoptosis as compared with the other groups in MCF-7 cells. The cellular uptake study revealed that this LNs were more readily uptaken by MCF-7 cells and M2 macrophages *in vitro*. Additionally, the targeted effect of PTX/DHA-FA-LNs was aided by FA receptor-mediated endocytosis, and its cytotoxicity was proportional to the cellular uptake efficiency. The anti-tumor efficiency results showed that PTX/DHA-FA-LNs significant inhibited tumor volume growth, prolonged survival time, and reduced toxicity when compared with the other groups. These results indicated that DHA increases the sensitivity of tumor cells and tumor-associated macrophages (ATM2) to PTX, and synergistic effects of folate modification in breast cancer treatment, thus PTX/DHA-FA-LNs may be a promising nanocarrier for breast cancer treatment.

## 1. Introduction

Breast cancer is a common type of cancers in women, with high morbidity and mortality (Siegel et al., 2020; Sung et al., 2021). Some recent studies reported that the breast cancer rates tends to be younger (Gohler et al., 2017; Cardoso et al., 2019; Siegel et al., 2020). Chemotherapy is still a common treatment strategy for breast cancer in clinical. However, the side effects caused by chemotherapy, such as the toxicity to normal tissues, are the main factors that limit its clinical application (Ponde et al., 2019). Additionally, it is become a serious concern of the development of drug resistance with chemotherapeutics (Wu et al., 2014). To meet these challenges such as improving its efficacy and bio-safety in the treatment of breast cancer, it is urgently required to develop a suitable drug delivery system (DDS) for the effective delivery of the anti-cancer drugs to the tumor tissue.

Paclitaxel (PTX) is extracted from the bark and wood of Pacific Yew and is widely used in the treatment of various types of tumors in clinical, such as breast, pancreatic, cervical, ovarian, and other cancer (Untch et al., 2016; Gawde et al., 2018). However, it is hamper PTX clinical efficacy for its several limitations (Franco et al., 2019). For example, PTX is not sensitive to tumor tissues while have adverse side effects on normal tissues, including cardiovascular toxicity, gastrointestinal side effects, lung toxicity, and bone marrow suppression (Tang et al., 2020). In addition, the another problem of PTX is the poor water solubility which limits its practical clinical application (Yao et al., 2017). To against the side effects and reverse the resistance of PTX, the design of PTX-based nanocarriers can improve the selectivity and stability of the drug in the tumor microenvironment (Yao et al., 2017; Tang et al., 2020). The PTX-based nanocarriers can also prevent its degradation, alter biodistribution, and enhance pharmacokinetic profile ultimately reducing its accumulation and toxicity in the normal tissue (Shen et al., 2012; Khalifa et al., 2019). In addition, nanocarriers can concentrate on tumor tissues via the effect of permeability and retention (EPR), and ultimately lead to superior clinical efficacy (Park et al., 2019).

Recently, some studies have reported that PTX and appropriate adjuvants were co-encapsulated in nanocarriers to enhance the anti-tumor activity of PTX and reduce toxicity (Khalifa et al., 2019; Qin et al., 2020). DHA, which was known as an omega-3 polyunsaturated fatty acid, has been proved to enhance the cytotoxic activity of several anti-tumor drugs, especially by producing oxidative damage and increasing the sensitivity of cancer cells (Maheo et al., 2005; Siddiqui et al., 2011; Fabian et al., 2015). DHA can inhibit cell metabolism in a dose-dependent manner, and inhibit the proliferation and growth of breast cancer *in vivo* and *in vitro* (Mouradian et al., 2015; Rescigno et al., 2016). In addition, DHA exerted a cardiovascular protective effect when combined with PTX (Adkins & Kelley, 2010; Serini et al., 2017). Hence, we proposed that the co-delivery of PTX and DHA in lipid nanoemulsions (LNs) could be an effective strategy to fight breast cancer for the passive tissue targeting, the protective of adverse effects and the ability to sensitize cancer cells.

In recent decades, nanotechnology is an upcoming and rapidly expanding field, which affects our lives tremendously in different fields, especially in medicine (Yu et al., 2021; Zheng et al., 2021). Some studies have been devoted to the preparation of targeted DDS to provide selective and sensitive transportation of highly cytotoxic anti-cancer. Tumor penetration is important for effectively tumor targeting DDS (Hu et al., 2021). These DDS could improve pharmacokinetic and increase therapeutic efficiency of anti-cancer agents while decreasing normal tissue toxicity (Oroojalian et al., 2018; Ren et al., 2021). To fabricate the DDS for cancer treatment, a number of ligands were attached to the nanocarriers to enhance the targeting of drugs to cancer cells, such as endothelial growth factors (EGFs), carbohydrates, amino acids, aptamers, as well as folic acid (FA) (Levy-Nissenbaum et al., 2008; Alibolandi et al., 2015; Kumari et al., 2016). FA, also called vitamin B6, is a water-soluble vitamin that plays an essential role in cellular growth and proliferation, especially during the period of the fetus. On the other hand, folate receptors are highly expressed on the surface of cancer cells. Therefore, FA could be a potent ligand of nanocarriers because of its high affinity to its receptor (Alibolandi et al., 2017; Muhamad et al., 2018; Jin et al., 2020).

**Figure 1.**
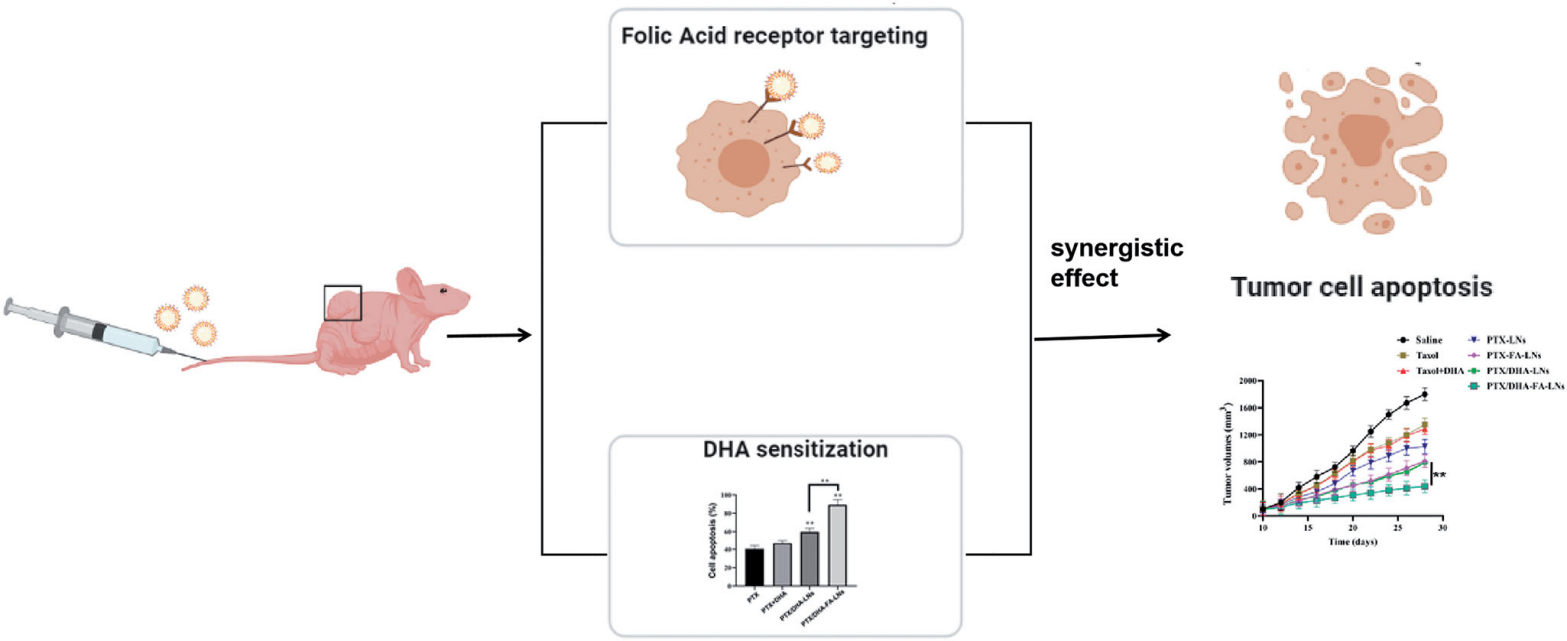
Co-delivery of PTX and DHA by targeting lipid nanoemulsions for breast cancer therapy.

Herein, we fabricated the assembly of LNs with FA co-modified loading PTX and DHA (PTX/DHA-FA-LNs) (Figure 1). *In vitro* studies were performed to verify the synergistic effects, cytotoxicity, apoptosis, and cellular uptake of this LNs in MCF-7 cells. Then, the anti-cancer activity and preliminary toxicity of the PTX/DHA-FA-LNs were evaluated in MCF-7 cells xenografted BALB/c nude mouse.

## 2. Materials and methods

### 2.1. Materials

PTX (CAS: 33069-62-4) was purchased from Kangnuo Chemical Co., LTD (Xi’an, China). Docosahexaenoic acid (DHA) as triglyceride was obtained from Croda Inc. (Edison, NJ). DSPE-PEG2000-FA (CAS: 1236288-25-7) was obtained from Ponsure Biopharma Co., Ltd. (Shanghai, China). Cholesterol (CHOL) (CAS: 57-88-5) and egg phosphatidylcholine (EPC) (CAS: 97281-44-2) were purchased from Sinopharm Chemical Reagent (Shanghai, China). Soybean oil (CAS: 8001-22-7) was purchased from Beiya Medical Oil Co., Ltd. (Tieling, China). Dulbecco’s modified Eagle’s medium (DMEM), penicillin/streptomycin solution, and fetal bovine serum (FBS) were obtained from Gibco BRL (Grand Island, NY). The water used in this experiment was deionized water.

### 2.2. Cell lines and animals

MCF-7 cells and RAW 264.7 cells were purchased from the Chinese Academy of Sciences (Shanghai, China) and grown with DMEM medium containing 10% FBS and 1% (v/v) penicillin/streptomycin and incubated in a humidified incubator with 5% CO_2_ at 37 °C.

BALB/c nude female mice of 3–4 weeks were obtained from Animal Center of Anhui Medical University (Hefei, China). All mice were fed following the directions of the China Council on Animal Care. All experimental protocols described in this study were approved by the Ethics Committee of Anhui Medical University.

### 2.3. Preparation of PTX/DHA-LNs and PTX/DHA-FA-LNs

In brief, a mixed solution was formed by PTX, DHA, EPC, CHOL, with DSPE-PEG2000-FA dissolved in 6 mL chloroform, and then the organic solvent was removed by rotary evaporation at 45 °C, forming a thin film on the round bottom flask. The vacuum was retained for 0.5 hour to ensure complete removal of any solvent. Then added an appropriate amount of water to the lipid film for hydration at 37 °C. After that, PTX/DHA-FA-LNs were obtained by using a microfluidizer. At the same time, the PTX/DHA-LNs without DSPE-PEG2000-FA, were also prepared by the above methods.

### 2.4. Characterization of PTX/DHA-LNs and PTX/DHA-FA-LNs

The particle size and zeta position (ZP) of the PTX/DHA-LNs and PTX/DHA-FA-LNs were measured by Zetasizer Nano-ZS90 (Malvern Instruments, Malvern, UK). All data were obtained in triplicate. The morphology of the LNs was measured using transmission electron microscope (TEM) with negative staining method. Briefly, the LNs were diluted in ultrapure water 50-times before the analyses and placed on a Formvar-copper grid. Then the grid was stained with 2% (w/v) aqueous uranyl acetate, dried at room temperature overnight and then observed using a TEM (Tecnai G2-12, 120 kV, FEI, Hillsboro, OR).

### 2.5. Determination of entrapment efficiency (EE) and drug loading (DL)

The LNs solution were diluted with 70% (v/v) ethanol. The PTX concentration was measured by using UV–vis spectrophotometer (Jasco V-750, Tokyo, Japan) at a wavelength of 227 nm at a PTX concentration range of 1–50 μg/mL. EE and DL of PTX loaded LNs were calculated by using the following equations:

EE (%) = (amount of PTX in LNs/amount of PTX fed) × 100

DL (%) = (amount of PTX in LNs/amount of PTX loaded LNs) ×100

### 2.6. *In vitro* PTX release

The PTX release study from PTX/DHA-FA-LNs was performed using the dynamic dialysis method *in vitro*. The standard of PTX was dissolved in ethanol and castor oil to being a control solution. Meanwhile, PTX/DHA-FA-LNs and PTX/DHA-LNs were dissolved in 2 mL deionized water and filled into dialysis bags with molecular weight of 3.5 kDa. The dialysis bags were placed into two centrifugal tube respectively which contain 40 mL PBS with Tween-80 (0.5%, w/w) and incubate at 37 °C with mild waggle (100 rpm) subsequently. Then, 3 mL of incubation medium was collected at a fixed time and the same volume of freshly preheated medium was added (0.5 h, 1 h, 2 h, 4 h, 6 h, 8 h, 10 h, 24 h, 36 h, 48 h, and 80 h). Last, the collected medium was centrifuged at 4 °C and 13,000 rpm for 30 min. The supernatant was then collected and was analyzed by HPLC.

### 2.7. Cell viability assay

Cell viability assay of PTX, PTX + DHA, PTX/DHA-LNs, and PTX/DHA-FA-LNs against MCF-7 cell line was assessed by MTT assay *in vitro*. For MTT assay, briefly, 100 μL of cell suspension with a density of 1 × 10^4^ cells/well was seeded and cultivated for 24 h in a 96-well plate. Then, the cells were incubated for 48 h with the treatment of different formulations. Thereafter, the MTT assay was performed according to the reagent manufacturer’s instructions. The absorbance of each place was measured by microplate reader (Multiskan EX, Thermo Scientific, Waltham, MA) at 490 nm.

### 2.8. Apoptosis assay by flow cytometry

Apoptosis experiment was performed with Annexin V-FITC and propidium iodide (PI) double staining method by flow cytometry experiment. MCF-7 cells were seeded in a 24-well plate at 2.5 × 10^5^ each plate and cultured for 24 h. Then, the cells were added with PTX, PTX/DHA, PTX/DHA-LNs, and PTX/DHA-FA-LNs for 72 h. After co-incubation, the cells were washed twice with PBS, trypsinized, and then tested using apoptosis detection kit I following the manufacturer’s protocol. Then, apoptosis was analyzed by using FACS Calibur (Becton Dickinson, San Diego, CA) under flow cytometry. The experiments were performed in duplicate and the results were analyzed using FlowJo software (Becton Dickinson, San Diego, CA).

### 2.9. Cellular uptake by confocal microscopy and regulation of macrophage polarization *in vitro*

To evaluate cellular uptake of the LNs, hydrophobic fluorescent dye, DiD was loaded instead of the PTX. MCF-7 cells and M2 macrophages (RAW 264.7 were stimulated with IL-4 (20 ng/mL) for 12 h) were seeded into 24-well plates at a density of 1.0 × 10^4^ per well and cultured in 500 μL medium for 24 h before the experiment. Then, the medium was discarded and the cells were treated with DiD, DiD + DHA, DiD/DHA-LNs, and DiD/DHA-FA-LNs for 0.5 h and 2 h. After co-incubation, the cells were washed three times with cold PBS, fixed with 10% formaldehyde solution for 20 minutes, and then washed three times with PBS again, and stained nuclear DNA with 0.1 μg/mL DAPI. Then the confocal plate was analyzed with LSM780 confocal laser scanning microscope (Zeiss, Oberkochen, Germany).

RAW264.7 cells were transformed into M1-type macrophages (M2) under stimulation with LPS- and IFN-γ, which served as a positive control. We divided the cells into five groups: PBS group, PTX group, PTX + DHA group, PTX/DHA-LNs group, PTX/DHA-FA-LNs, and M2-type macrophages (IL-4-stimulated RAW 264.7 cells) incubated for 3 h. M1 and M2 macrophages were labeled with CD86 antibody and CD206 antibody, respectively. The ability of M1 macrophages to transform into M2 macrophages was investigated by laser confocal microscopy.

### 2.10. *In vivo* anti-tumor activity of PTX/DHA-FA-LNs

To evaluate the efficacy of the synthesized nanoemulsion, BALB/c nude mice transplanted with MCF-7 cells were used as breast cancer model. The ectopic tumor mice (weight of 18–20 g) model was established by subcutaneously injecting 80 μL of MCF-7 cells suspension containing 5 × 10^5^ cells into the right flank of the mice. After 15 days inoculation and tumor volume reached about 100 mm^3^, mice were then treated with PTX/DHA-FA-LNs, PTX/DHA-LNs, PTX-FA-LNs, PTX-LNs, PTX + DHA, and PTX by injecting via tail vein (six mice per group). A single dose of saline injection was used as a negative control. Systemic toxicity was assessed by monitoring tumor volumes, mice weight change during 30 days post-injections and survival rate during 100 days. The tumor volume calculation formula is as follows:

Tumor volume=0.5×length×width×height.

### 2.11. Safety evaluation

All mice were sacrificed and heart, liver, spleen, lung, and kidney samples were collected and processed for histological analysis. Samples of tissue (approximately 100 mg) were fixed for one day with 4% formalin, then dehydrated and embedded in paraffin. The tissues were also stained with hematoxylin and eosin (H&E). The image acquisition and analysis system were obtained by using an inverted fluorescence microscope.

### 2.12. Statistical analysis

All data were expressed as mean ± standard deviation (SD). Statistical comparisons were evaluated by the analysis of variance between two groups, and means comparisons were performed by Student’s *t*-test. All statistical analysis were shown using GraphPad Prism software (La Jolla, CA).

## 3. Results

### 3.1. MTT assay to screen the optimal concentration ratio of PTX and DHA

**Figure 2.**
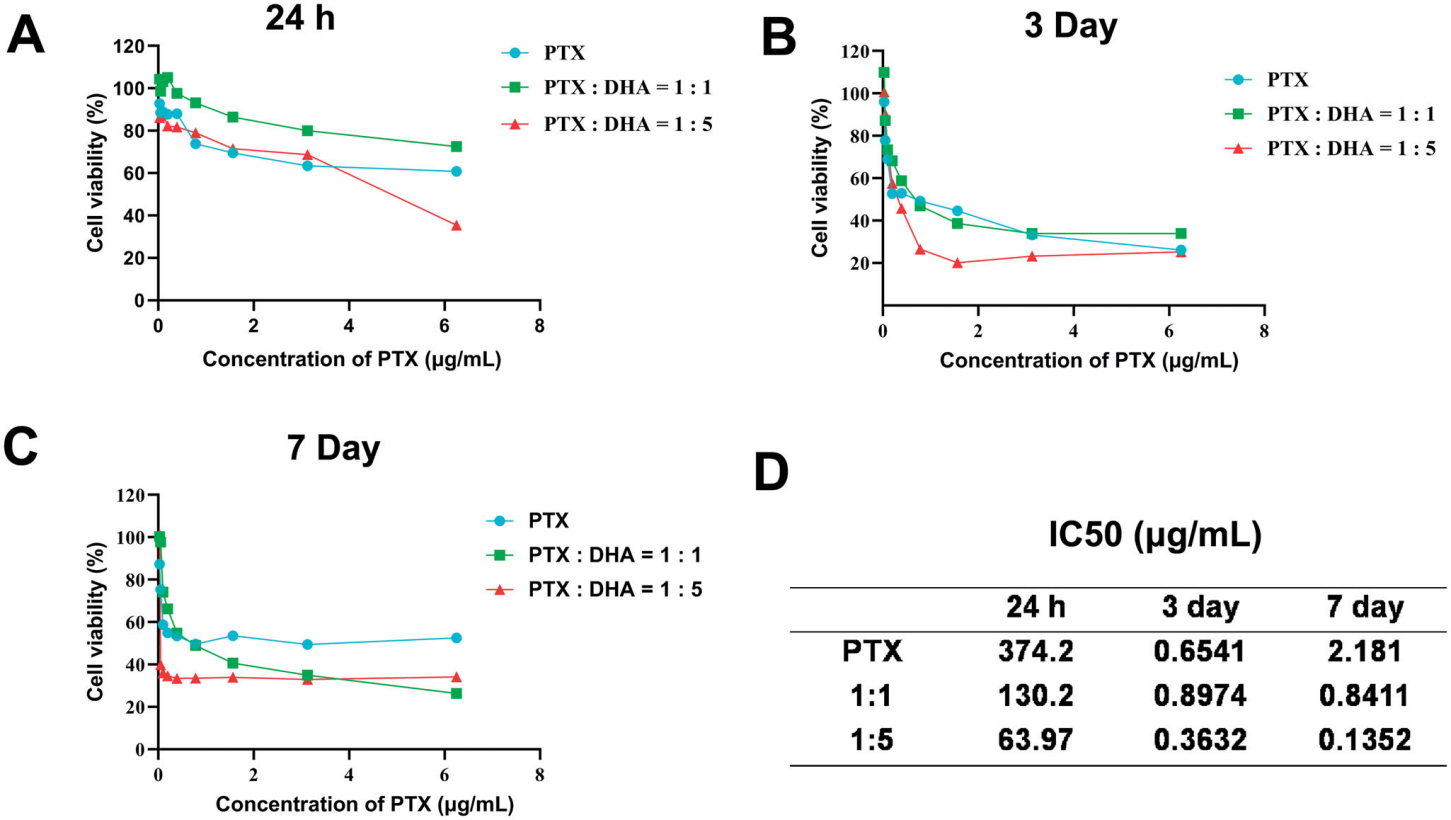
(A–C) Cell toxicity of PTX and DHA in different concentration ratios to MCF-7 cells. (D) The IC_50_ of PTX and DHA in different ratios over time. Data were represented as mean ± SD (*n* = 3).

Viabilities of MCF-7 cells after treatments with PTX, PTX with DHA (1:1), and PTX with DHA (1:5) were evaluated by MTT assay. As demonstrated in Figure 2(A–C), when PTX was combined with DHA (1:1 and 1:5) compared to PTX treatment alone, a slight decrease was observed in cell viability. To clarify the possible synergy between PTX and DHA, the data were analyzed with CalcuSyn software (Figure 2(D)). The result indicated that PTX combined with DHA (1:5) had the best synergy to MCF-7 cells (IC_50_=0.1352 μg/mL) in seven days. In this experimental study, we chose PTX and DHA at a ratio of 1:5 in the following experiments.

### 3.2. Characterization of PTX/DHA-LNs and PTX/DHA-FA-LNs

**Figure 3.**
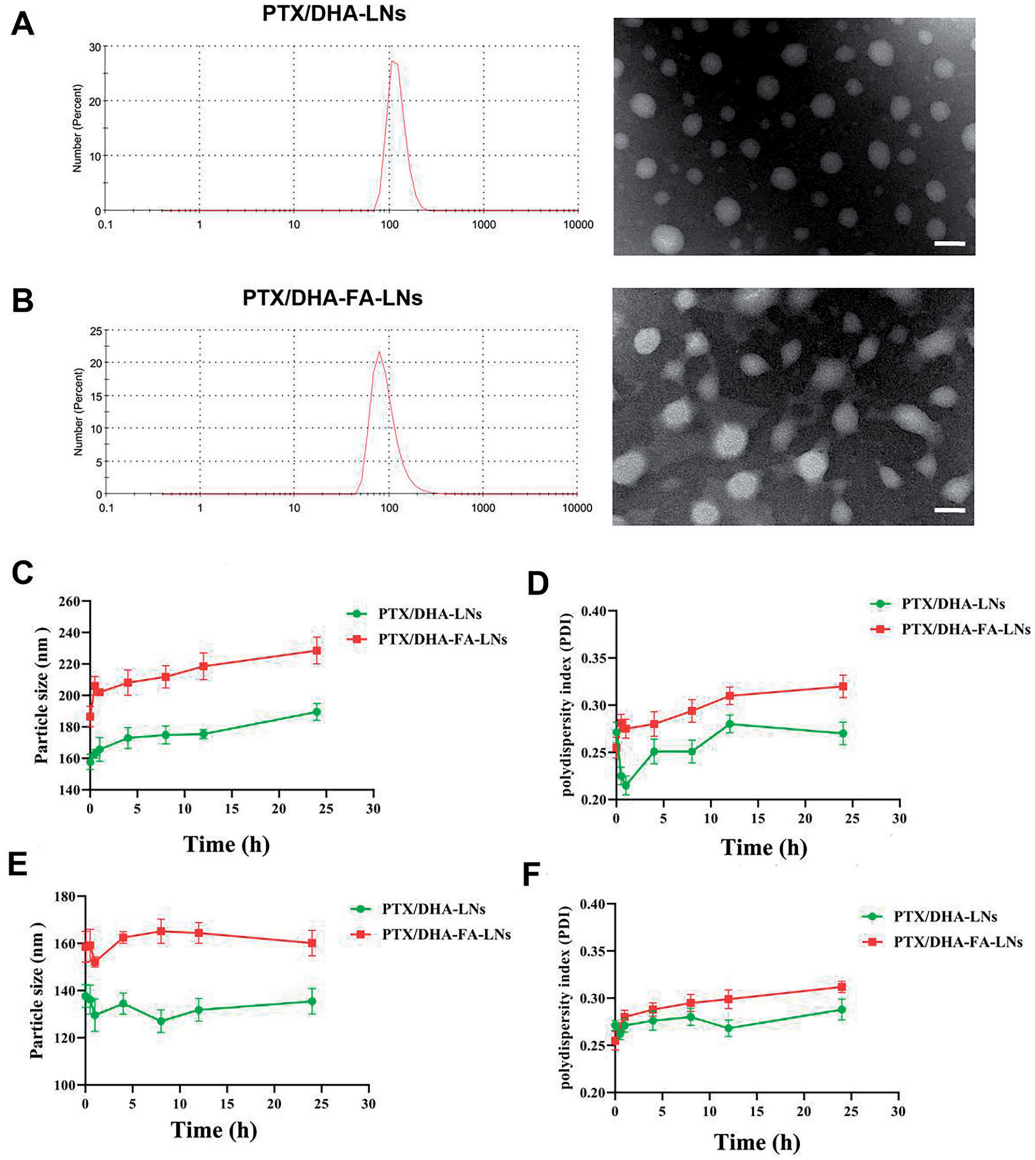
*In vitro* stability of PTX/DHA-LNs and PTX/DHA-FA-LNs. (A, B) The changes of the particle size and PDI of LNs in PBS over time. (C, D) The changes of the particle size and PDI of LNs in serum over time. Scale bar represents 200 nm.

In this study, two different of LNs loaded with PTX/DHA were developed. Table 1 shows the mean size, PDI, DL and EE of PTX/DHA-LNs and PTX/DHA-FA-LNs. The mean particle size and PDI of the PTX/DHA-LNs and PTX/DHA-FA-LNs were 157.7 ± 4.2 nm, 0.271 ± 0.009 and 186.6 ± 4.9 nm, 0.255 ± 0.011, respectively. The two nanoemulsion showed a uniformly size distribution with PDI < 0.3, indicating narrow size dispersion (Figure 3(A,B)). There is no significant difference between PTX/DHA-LNs and PTX/DHA-FA-LNs in diameters. Additionally, there is also no significant difference between PTX/DHA-LNs and PTX/DHA-FA-LNs in DL and EE (*p*> .05).

**Table 1.** Characterization of PTX/DHA-LNs and PTX/DHA-FA-LNs.

	Particle size (nm ± SD)	PDI ± SD	DL (%±SD)		EE (%±SD)	
			PTX	DHA	PTX	DHA
PTX/DHA-LNs	157.7 ± 4.2	0.271 ± 0.009	4.8 ± 0.17	24.5 ± 0.44	97.3 ± 4.9	90.7 ± 5.3
PTX/DHA-FA-LNs	186.6 ± 4.9	0.255 ± 0.011	5.2 ± 0.23	26.1 ± 0.56	96.2 ± 5.1	92.3 ± 6.2

PDI: polydispersity index; DL: drug loading; EE: encapsulation efficiency.

Data were represented as mean ± SD (*n* = 3).

### 3.3. The stability of these LNs *in vitro*

The stability of the prepared PTX/DHA-LNs and PTX/DHA-FA-LNs was evaluated in PBS and serum to investigate the effect of LNs aggregation and size changes (Figure 3(C–F)). The obtained results indicated that both the PTX/DHA-LNs and PTX/DHA-FA-LNs systems were stable both in PBS and serum during 24 h incubation. In addition, the prepared PTX/DHA-FA-LNs showed larger particle size and PDI both in PBS and serum in comparison with PTX/DHA-LNs, which could be attributed to the LNs with FA co-modified leads to increased particle size and PDI.

### 3.4. *In vitro* drug cumulation release

**Figure 4.**
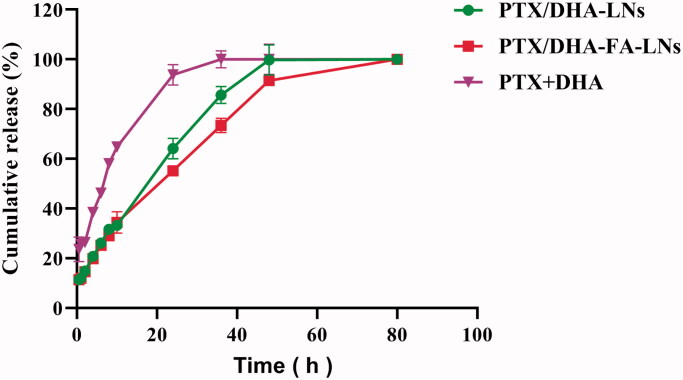
*In vitro* drug release profiles of PTX from PTX + DHA, PTX/DHA-LNs, and PTX/DHA-FA-LNs.

Drug release was measured in phosphate buffer (Figure 4) to simulate *in vivo* conditions. The results showed that drug release is controllable and continuous, and there is no burst effect. In this regard, PTX/DHA-FA-LNs group drug cumulation release assay showed that 100% of the drug was released within 48 hours. Release profiles of PTX from PTX/DHA-FA-LNs and PTX/DHA-LNs systems showed no significant differences (*p*> .05).

### 3.5. Cytotoxicity

**Figure 5.**
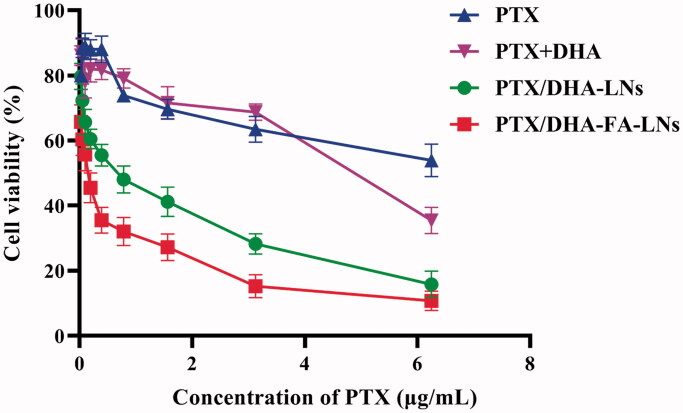
*In vitro* cell viability of PTX, PTX + DHA, PTX/DHA-LNs, and PTX/DHA-FA-LNs in MCF-7 cells after 24 h of incubation.

To elucidate the efficacy of prepared LN in transportation of PTX into the cells, MTT assay was performed in MCF-7 cell lines. As shown in Figure 5, the result indicated that both PTX/DHA-FA-LNs and PTX/DHA-LNs showed greater cytotoxicity compared to PTX and PTX + DHA solution in MCF-7 cells after 24 h treatment. Moreover, PTX/DHA-FA-LNs (IC_50_=0.391 µg/mL) showed higher inhibitory effect compared to PTX/DHA-LNs (IC_50_=0.536 µg/mL). These results indicated that DHA and FA can enhance the cytotoxicity of PTX to tumor cells.

### 3.6. Cellular uptake in MCF-7cells

**Figure 6.**
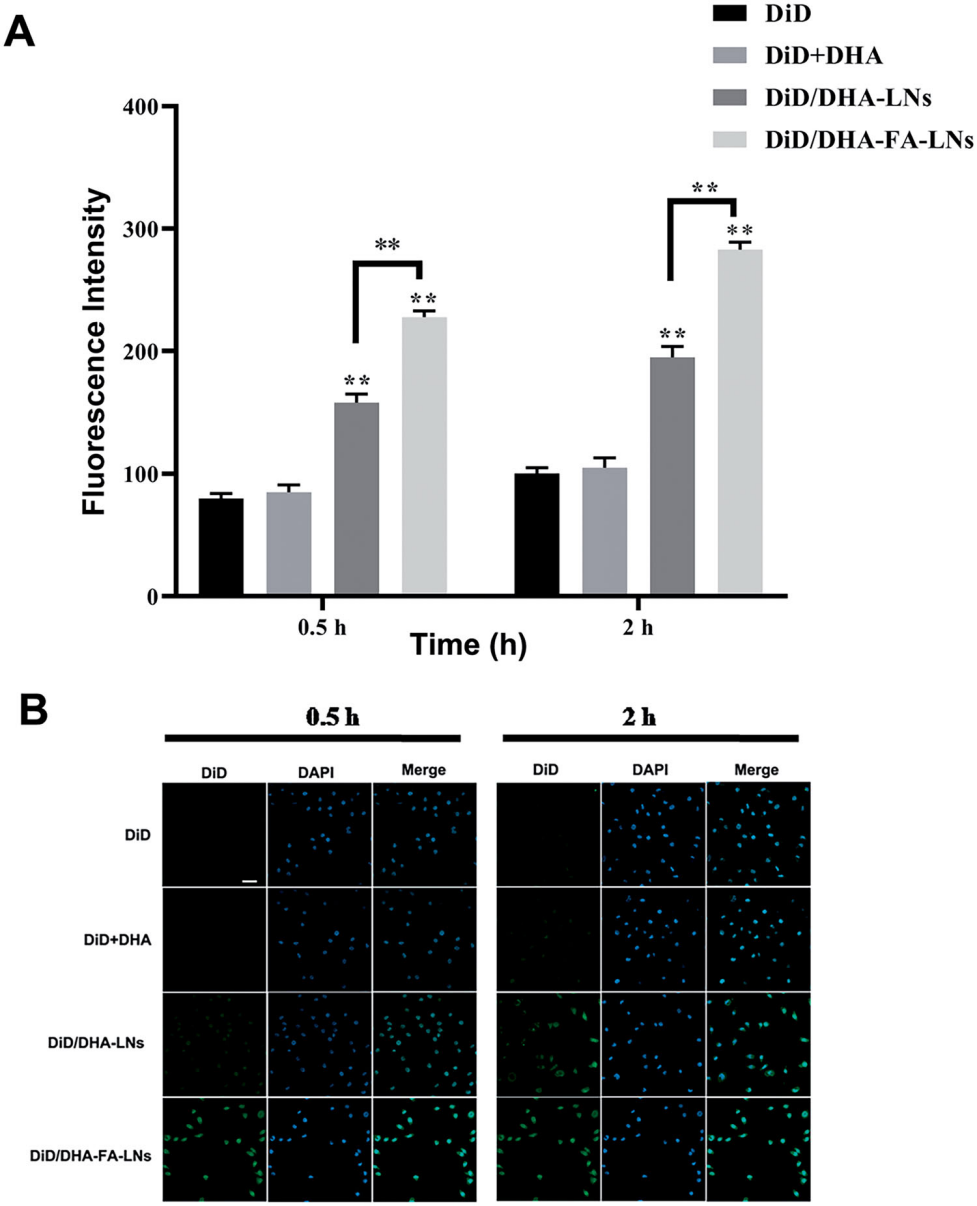
(B) Representative confocal laser scanning microscopy images of MCF-7 cells after incubation with PTX/DHA-FA-LNs and PTX/DHA-LNs for 0.5 h and 2 h. DAPI stains for cell nuclei (blue); DiD represents PTX (green). Scale bar represents 30 µm. (A) Cell uptake efficiency in MCF-7 was quantified by FACS analysis. Data represent means ± SD (*n* = 3) (**p< .01).

To clarify the cellular uptake efficiency in MCF-7 cells of PTX/DHA-LNs and PTX/DHA-FA-LNs, DiD was used instead of PTX which is a near-infrared fluorescence probe. DiD-loaded nanoemulsion such as DiD/DHA-LNs and DiD/DHA-FA-LNs was synthesized following the method as described in section ‘Materials and methods’. The cellular uptake of DiD, DiD/DHA, DiD/DHA-LNs, and DiD/DHA-FA-LNs in MCF-7 cells is represented in Figure 6. The MCF-7 cells were incubated with DiD, DiD/DHA, DiD/DHA-LNs, and DiD/DHA-FA-LNs for 0.5 h and 2 h. According to the results, both DiD/DHA-FA-LNs (228.3 ± 5.6 in 0.5 h and 283.8 ± 6.1 in 2 h) and DiD/DHA-LNs (158.1 ± 7.2 in 0.5 h and 195.5 ± 9.3 in 2 h) showed time-dependent enhancement in fluorescence intensity in MCF-7 cells (Figure 6(A)). Besides, cells treated with DiD/DHA-FA-LNs (228.3 ± 5.6 in 0.5 h and 283.8 ± 6.1 in 2 h) showed intense fluorescent intensity as compared to the other groups, indicating that PTX and DHA loaded with FA decorated in LN were more easily uptake in MCF-7 cells (Figure 6(B)).

### 3.7. Cell apoptosis

**Figure 7.**
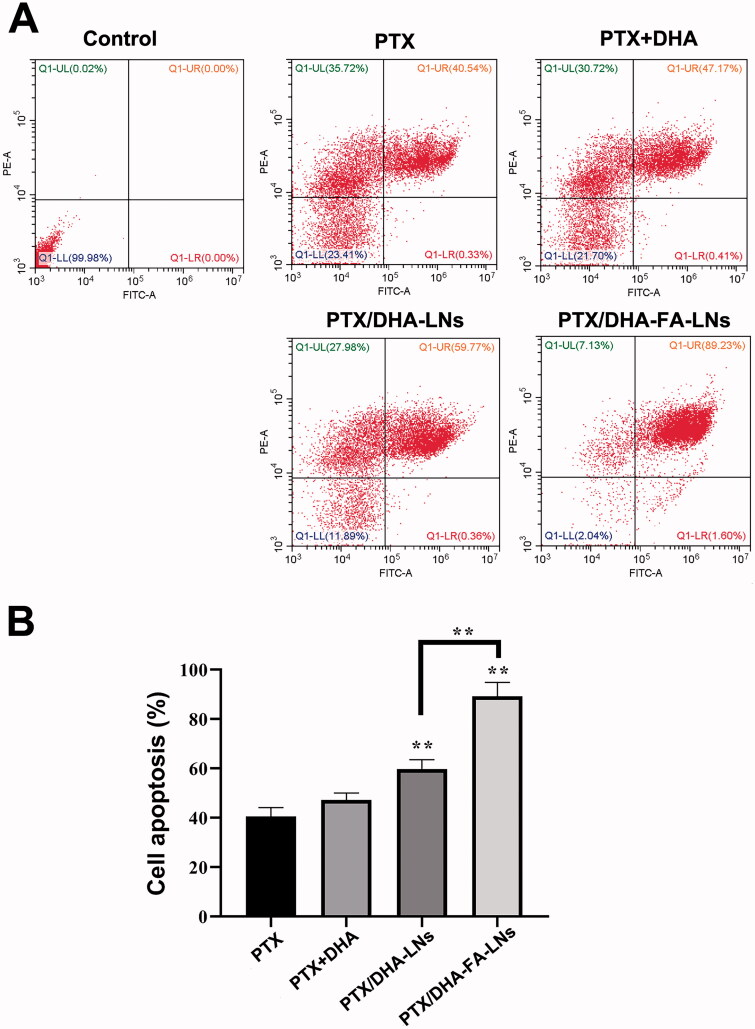
(A) Cell apoptosis and necrosis were analyzed by flow cytometry using annexin V-FITC in combination with PI in MCF-7 cells. (B) The quantification of apoptotic and necrotic cell percentages after treatment with different formulations in MCF-7 cells (**p< .01).

The cell apoptosis assay was carried out in MCF-7 cells using flow cytometry and the results are showed in Figure 7(A,B). The group of PTX/DHA-FA-LNs exhibited higher apoptosis rate (89.23%±5.66%) as compared to the group of PTX/DHA-LNs (59.77%±3.56%, *p*<.01), PTX-LNs (47.17%±2.88%, *p*< .01), and PTX alone (40.54%±3.58%, *p*< .01). The PTX/DHA-LNs group also showed a significant difference as compared to the PTX/DHA and PTX alone groups (*p*< .01). The results indicated that the significant cytotoxicity of PTX/DHA-FA-LNs to MCF-7 cells may be through the apoptosis pathway. The cell apoptosis results showed that there was no significant difference between the PTX/DHA group and the PTX alone group (*p*> .05).

### 3.8. Cellular uptake in M2 macrophage cells and regulation of macrophage polarization *in vitro*

**Figure 8.**
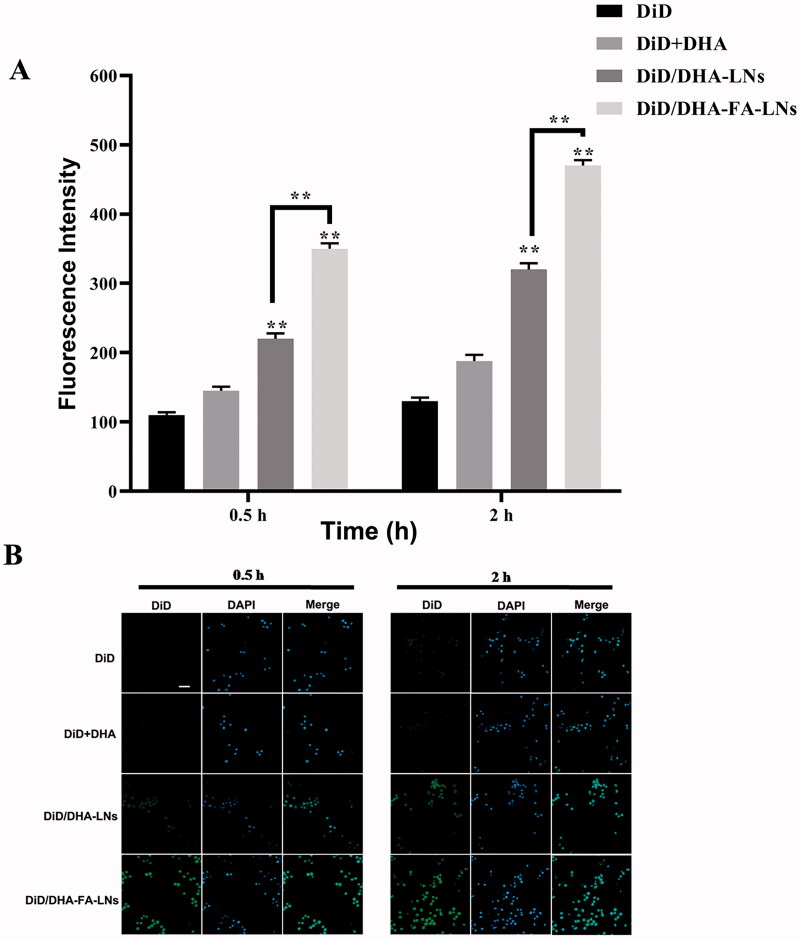
(B) Representative confocal laser scanning microscopy images of M2 macrophage cells after incubation with DiD/DHA-FA-LNs and DiD/DHA-LNs for 0.5 h and 2 h. DAPI stains for cell nuclei (blue); DiD represents PTX (green). Scale bar represents 30 µm. (B) Cell uptake efficiency in M2 macrophage was quantified by FACS analysis. Data represent means ± SD (*n* = 3) (**p< .01).

It was verified that the immune system can constantly check and remove cancer cells, to prevent and inhibit the development of cancer (Wang et al., 2019). M2 macrophages were chosen in the cellular uptake efficiency experiment of PTX/DHA-FA-LNs, which is a crucial immune cell type in the tumor microenvironment. According to results, both PTX/DHA-FA-LNs (350.1 ± 8.6 in 0.5 h and 473.0 ± 8.7 in 2 h) and PTX/DHA-LNs (321.2 ± 9.5 in 0.5 h and 321.2 ± 9.5 in 2 h) showed the fluorescence signal in M2 macrophages increased in a time-dependent manner. At all given time points, PTX/DHA-FA-LNs (350.1 ± 8.6 in 0.5 h and 473.0 ± 8.7 in 2 h) exhibited significantly higher cellular uptake efficiencies as compared to PTX/DHA-LNs (*p*< .01) in M2 macrophages (Figure 8(A,B)). The results indicated that the preparation of PTX/DHA-FA-LNs could be specifically ingested by M2 macrophages, thereby may inhibit tumor growth at the immune system.

**Figure 9.**
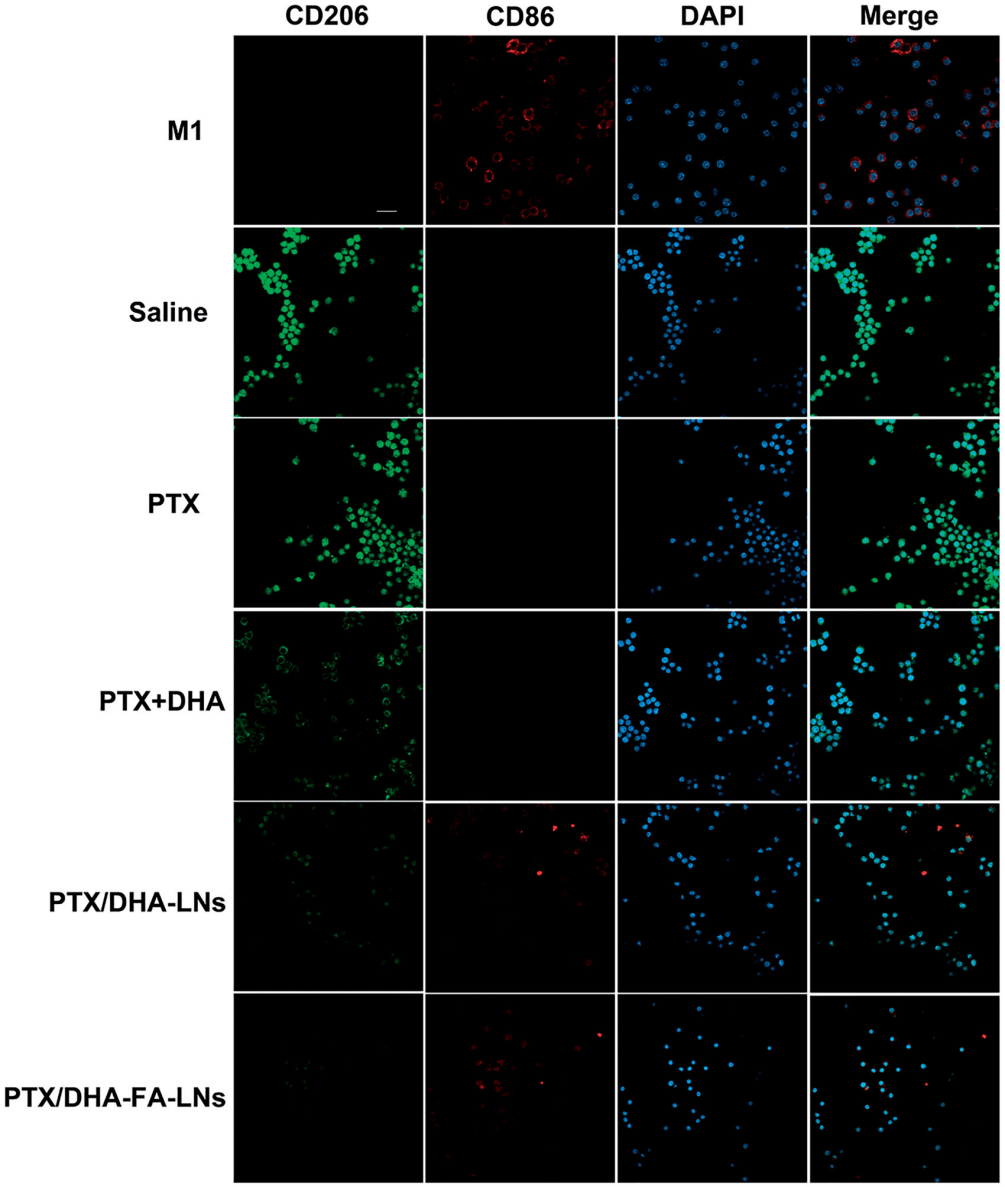
Confocal laser scanning microscopy images of macrophages after treatment with PBS, PTX, PTX + DHA, PTX/DHA-LNs, and PTX/DHA-FA-LNs. Cell nuclei were stained with DAPI (blue), CD86 fluorescence displayed in red and CD206 fluorescence displayed in green. Scale bar represents 20 μm. Data represent mean ± SD (*n* = 3).

Confocal laser scanning microscopy showed that the number of M2 macrophages in saline group was the highest (Figure 9). In the given group, the number of M2 macrophages gradually decreased and the number of M1 macrophages gradually increased in PTX, PTX + DHA, and PTX/DHA-LNs groups. M1 macrophages in the PTX/DHA-FA-LNs group were similar to those in the positive group. *In vitro* results confirmed that PTX/DHA-FA-LNs could promote the transformation of M2 macrophages into M1 macrophages and delayed the progression of tumor.

### 3.9. Anti-tumor efficacy *in vivo*

**Figure 10.**
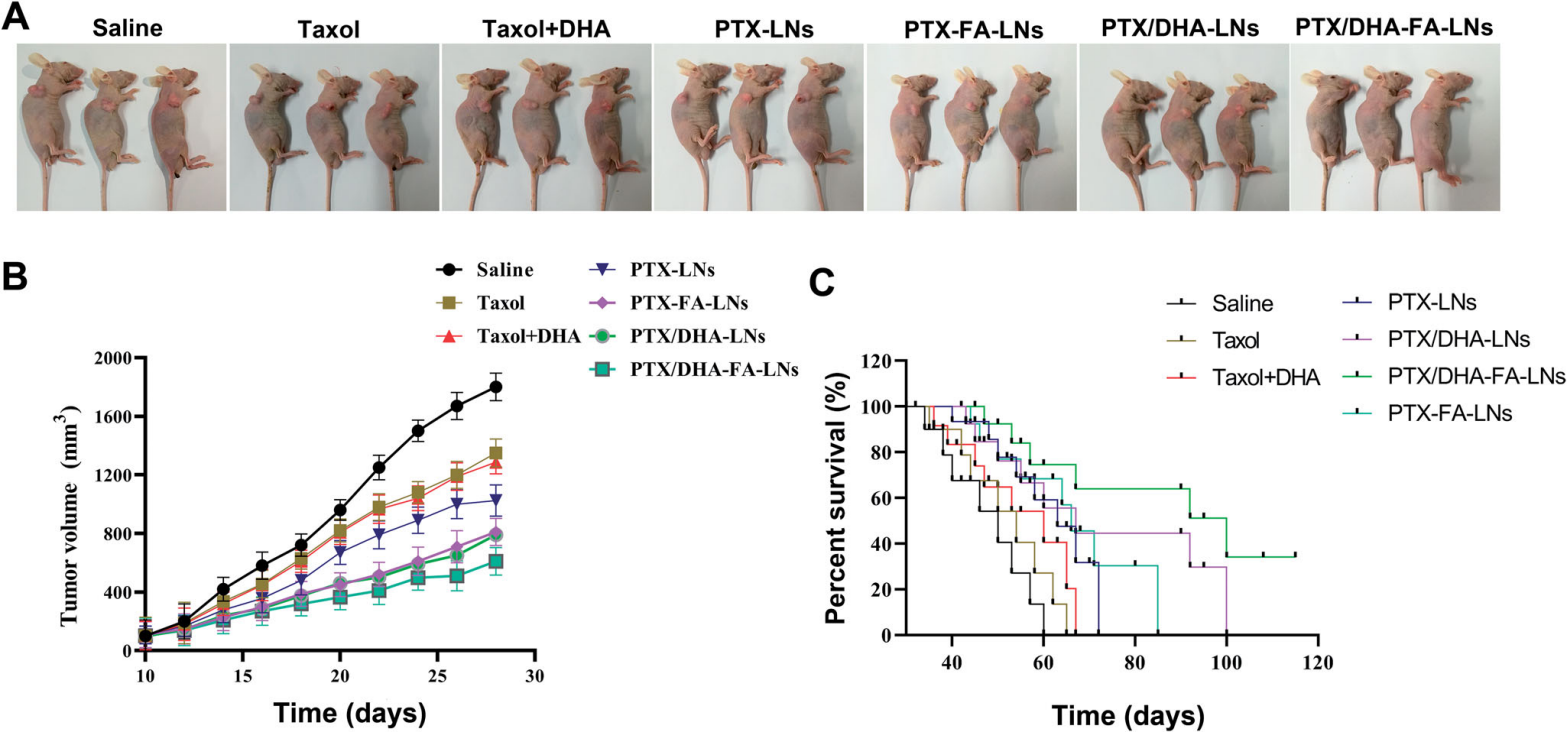
(A) *In vivo* therapeutic efficiency of a single-dose intravenous injection of different formulations to inhibit tumor growth in BALB/c mice bearing MCF-7 tumors (*n* = 3). (B) Average tumor volumes after treatment over the investigative period (*n* = 3). (C) The survival rate of mice in each group (**p< .01).

The *in vivo* therapeutic effects of PTX and LNs were studied in nude mice with transplanted tumors of MCF-7 cells. The results exhibited that the tumor volume of mice grew rapidly in the saline group (Figure 10(A,B)). Conversely, the groups treated with treatment were showed slower tumor growth. On the other hand, the mice treated with PTX/DHA-LNs and PTX/DHA-FA-LNs exhibited significantly higher tumor growth inhibitory effects, treated with PTX/DHA-FA-LNs showed the best tumor growth inhibition, indicating that co-loaded DHA and FA decorated can synergistically enhance the anti-tumor activity of PTX nanoemulsions (Figure 10(B)). The mice survival curves are shown in Figure 10(C). The administration of PTX/DHA-FA-LNs significantly prolonged the survival time of nude mice as comparison to the other groups (100 days, *p*< .05), suggesting the potential clinical application of the PTX/DHA-FA-LNs.

### 3.10. Safety evaluation

**Figure 11.**
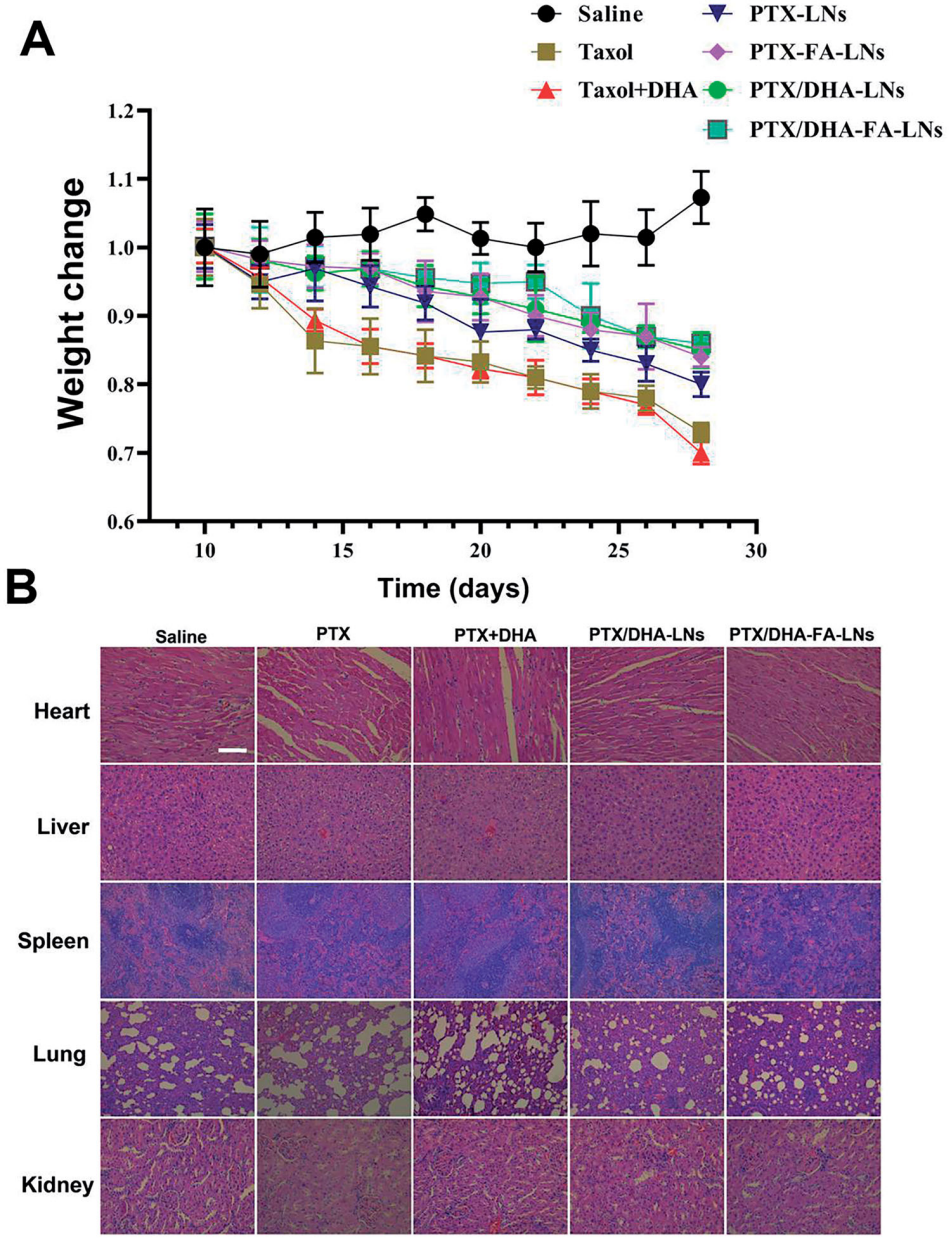
(A) Weight monitoring following a single-dose intravenous injection of different formulations to mice bearing MCF-7 tumor over time. (B) Morphology of different formulations groups. Tissues were isolated and stained with hematoxylin and eosin (H&E) for histopathological analysis. Scale bar represents 100 μm. Data represent means ± SD (*n* = 3).

During the treatment, no abnormalities in diet and behavior of nude mice in all groups were found, indicating that no serious adverse reactions occurred by the treatments of PTX/DHA-FA-LNs. The body weights of the mice were recorded every other day as shown in Figure 11(A). The results showed that there was no significant difference in the body weight of the nude mice in all groups at the beginning. In the treatment group, a continuous decline in the weight of the mice was observed, which could be caused by the toxic effects of PTX. In addition, the result showed that the administration of PTX/DHA-FA-LNs treatment effectively inhibited the weight loss of mice.

H&E staining was performed on tissue sections including heart, liver, spleen, lung, and kidney of mice to evaluate the toxicity of PTX/DHA-FA-LNs *in vivo*. The results showed that slight lesion in liver, spleen, and kidney was observed in all treatment groups (Figure 11(B)). On the other hand, significant pathological changes in the heart and lung tissues appeared in the mice given PTX and PTX/DHA treatment groups including balloon generation. As compared to the group treated with PTX/DHA-FA-LNs and PTX/DHA-LNs, discrete loss of striation was observed, and vacuoles and hyaline degeneration were rare, indicating greater protection against the heart and lung toxicity effects of PTX. Altogether, the results indicated only mild heart and lung toxicity by PTX/DHA-FA-LNs and PTX/DHA-LNs *in vivo*.

## 4. Discussion

Breast cancer has a high morbidity and mortality rate that seriously threatens the health and lives of women throughout the world (Ma et al., 2019). Chemotherapy, hormone therapy, surgery, and radiation therapy are usually treatment for breast cancer. However, chemotherapy is usually accompanied by high toxicity and has many side effects. In addition, many studies have reported that tumor resistance is another challenge for chemotherapy drugs. Recently, studies have proposed therapeutic treatment strategies that combines two or more drugs for the treatment of breast cancer and this treatment strategies are considered to have a better efficacy, while reducing their side effects, enhancing the efficacy, and improving targeting efficiency. However, these treatment strategies often fails to achieve of the desired efficacy due to the different pharmacokinetics, disparate release behavior and problems with tissue targeting of the respective drugs (Yuan et al., 2008). In this study, nanocarriers can co-encapsulate two or more drugs in DDS with a controlled manner, and can target tumor tissues through the EPR effect and ligand modified, showing significant superiority compared with traditional combination therapy.

Recently, LNs have become a competitive DDS that can optimally deliver drugs to tumor tissues in different routes (such as parenteral, ocular, oral, and transdermal) (Chen et al., 2010; Hazeldine et al., 2014; Cao et al., 2016; Sun et al., 2016; Dehaini et al., 2017). LNs are oil-in-water nanocarriers with a particle size range of 20–200 nm and it can encapsulate hydrophobic drugs in amphiphilic lipid core (Niu et al., 2018). In this experiment, we encapsulated PTX and DHA in LNs with FA decorating together, and evaluated the anti-tumor effect of PTX/DHA-FA-LNs.

The characterization of PTX/DHA-LNs and PTX/DHA-FA-LNs is shown in Table 1. The PTX/DHA-FA-LNs and PTX/DHA-LNs had a suitable particle size of <200 nm and PDI <0.3, indicating that LNs can passively target tumor tissues through the EPR effect (Albanese et al., 2012). In addition, the corresponding EE of PTX and DHA were 96.2% and 92.3%, respectively. In our experiment, the LNs exhibited suitable water solubility to satisfy the requirement of intravenous injection. Moreover, release studies *in vitro* showed that these LNs were stable in PBS and in serum, achieving the requirements of nanocarriers (Figure 3). It was observed that PTX/DHA-FA-LNs and PTX/DHA-LNs exhibited a stronger anti-tumor efficacy as compared to the free PTX and PTX/DHA *in vitro* (Figure 5), which might be because DHA and PTX are co-encapsulated with PTX in LNs. DHA has been proven to enhance the cytotoxicity of several anti-cancer drugs, especially the sensitivity to cancer cells and oxidative stress damage (Maheo et al., 2005; Mussi et al., 2013). On the other hand, DHA has been shown to effectively block the NF-κβ signaling pathway leading to tumor cell apoptosis (Wang et al., 2011). In our supplementary experiments, it was confirmed that the sensitivity and toxicity of nanoemulsions to tumor cells and macrophages are reduced without DHA (Figures S2 and S3). The folate receptor is highly expressed on the surface of many tumor cells, but is noticeably low in normal tissues (Assaraf et al., 2014; Fasehee et al., 2016; Monteiro et al., 2020). Then, FA seems to be the optimal ligand for DDS to target cancer cells. Therefore, co-delivery of PTX and DHA by LNs with FA decorating could exert stronger cytotoxicity to breast cancer cells as compared to the PTX alone. In addition, the cell uptake experiment showed that the fluorescence intensity of DiD/DHA-FA-LNs (228.3 ± 5.6 in 0.5 h and 283.8 ± 6.1 in 2 h) in MCF-7 cells was significantly higher than that in the DiD group (82.3 ± 4.7 in 0.5 h and 101.4 ± 5.0 in 2 h), which confirmed the previous study (Figure 6).

Testing the anti-tumor activity of PTX/DHA-FA-LNs *in vivo* is a prerequisite for potential clinical applications. Therefore, we further studied the efficacy of PTX/DHA-FA-LNs in MCF-7 xenograft nude mice *in vivo*. In our research, the application of PTX/DHA-FA-LNs significantly inhibited tumor growth and prolonged the survival time of nude mice as compared to other groups. This indicated that LNs can accumulate in tumor tissues through the EPR effect, which enhances the inhibitory effect of PTX on tumors. The intravenous injection of PTX/DHA-FA-LNs can effectively restrain the weight loss of mice compared with the continuous weight loss of the PTX group during the treatment period, indicated that the administration did not have serious side effects compared with PTX group. Our results revealed that the administration of PTX/DHA-FA-LNs exhibits better anti-tumor effect and low toxicity than PTX *in vivo* (Cao et al., 2020).

Apoptosis plays an important role in the occurrence and development of tumors (Wong, 2011; Mohammad et al., 2015). Chemotherapy drugs can induce extensive apoptosis of tumor cells by interfering with the growth, metabolism, and proliferation. Therefore, inducing cell apoptosis can be the most effective treatment to cancer. A flow cytometry experiment was performed to clarify the cell apoptotic effect of PTX/DHA-FA-LNs cells *in vitro*. Compared with other groups, the administration of PTX/DHA-FA-LNs showed the highest rate of apoptosis on MCF-7 cells, indicating that DHA can enhance the apoptosis of PTX on tumor cells (Figure 7). In supplementary experiments, the apoptosis rate of PTX-FA-LNs (69.23%±4.70%) was significantly lower than that of PTX/DHA-FA-LNs (89.23%±5.66%) (Figure S4). The reason is probably that DHA can block the NF-κβ signaling pathway, and DHA can also reverse the resistance of PTX (Zhang et al., 2011; Mussi et al., 2014).

The anti-tumor efficacy of the prepared PTX/DHA-FA-LNs was verified by both *in vitro* and *in vivo* experiments. However, more rigorous experiments are required to study the application of PTX/DHA-FA-LN *in vivo*. Moreover, it is very meaningful to clarify the anti-tumor effect and the mechanism of reversing PTX resistance of the administration DHA. Therefore, DDS designed with nanocarriers provides a solid foundation and effective strategy for clinical treatment of breast cancer.

## 5. Conclusions

LNs loading PTX and DHA with FA modified was successfully prepared showing better targeting effect and DHA increases the sensitivity of tumor cells and tumor-associated macrophages (ATM2) to PTX, and synergistic effects of folate modification in breast cancer treatment. Release experiment exhibited a controlled PTX release from LNs *in vitro*. *In vitro* cell analysis showed that co-delivery of PTX and DHA in FA-LNs has synergistic effects against MCF-7 cells and enhance cell apoptosis. *In vivo* studies have shown that this formulation can significantly inhibit the growth of tumors, but also reduce the mortality of mice, and reduce the weight loss and the toxicity to the organs. In conclusion, these results support the proposal that co-delivery of PTX and DHA in FA-LNs is considered as a promising strategy for breast cancer treatment.

## Supplementary Material

Supplemental Material

## Data Availability

The raw data cannot be shared at this time as the data also forms part of an ongoing study.

## References

[CIT0001] Adkins Y, Kelley DS. (2010). Mechanisms underlying the cardioprotective effects of omega-3 polyunsaturated fatty acids. J Nutr Biochem 21:781–92. 20382009 10.1016/j.jnutbio.2009.12.004

[CIT0002] Albanese A, Tang PS, Chan WC. (2012). The effect of nanoparticle size, shape, and surface chemistry on biological systems. Annu Rev Biomed Eng 14:1–16. 22524388 10.1146/annurev-bioeng-071811-150124

[CIT0003] Alibolandi M, Ramezani M, Sadeghi F, et al. (2015). Epithelial cell adhesion molecule aptamer conjugated PEG-PLGA nanopolymersomes for targeted delivery of doxorubicin to human breast adenocarcinoma cell line *in vitro*. Int J Pharm 479:241–51. 25529433 10.1016/j.ijpharm.2014.12.035

[CIT0004] Alibolandi M, Rezvani R, Farzad SA, et al. (2017). Tetrac-conjugated polymersomes for integrin-targeted delivery of camptothecin to colon adenocarcinoma *in vitro* and *in vivo*. Int J Pharm 532:581–94. 28935257 10.1016/j.ijpharm.2017.09.039

[CIT0005] Assaraf YG, Leamon CP, Reddy JA. (2014). The folate receptor as a rational therapeutic target for personalized cancer treatment. Drug Resist Updat 17:89–95. 25457975 10.1016/j.drup.2014.10.002

[CIT0006] Cao H, Dan Z, He X, et al. (2016). Liposomes coated with isolated macrophage membrane can target lung metastasis of breast cancer. ACS Nano 10:7738–48. 27454827 10.1021/acsnano.6b03148

[CIT0007] Cao X, Tan T, Zhu D, et al. (2020). Paclitaxel-loaded macrophage membrane camouflaged albumin nanoparticles for targeted cancer therapy. Int J Nanomedicine 15:1915–28. 32256068 10.2147/IJN.S244849 PMC7090179

[CIT0008] Cardoso F, Kyriakides S, Ohno S, et al. (2019). Early breast cancer: ESMO Clinical Practice Guidelines for diagnosis, treatment and follow-up. Ann Oncol 30:1194–220. 31161190 10.1093/annonc/mdz173

[CIT0009] Chen Y, Bathula SR, Yang Q, et al. (2010). Targeted nanoparticles deliver siRNA to melanoma. J Invest Dermatol 130:2790–8. 20686495 10.1038/jid.2010.222

[CIT0010] Dehaini D, Wei X, Fang RH, et al. (2017). Erythrocyte-platelet hybrid membrane coating for enhanced nanoparticle functionalization. Adv Mater 29:1606209. 10.1002/adma.201606209 PMC5469720 28199033

[CIT0011] Fabian CJ, Kimler BF, Hursting SD. (2015). Omega-3 fatty acids for breast cancer prevention and survivorship. Breast Cancer Res 17:62. 25936773 10.1186/s13058-015-0571-6 PMC4418048

[CIT0012] Fasehee H, Dinarvand R, Ghavamzadeh A, et al. (2016). Delivery of disulfiram into breast cancer cells using folate-receptor-targeted PLGA-PEG nanoparticles: *in vitro* and *in vivo* investigations. J Nanobiotechnol 14:32. 10.1186/s12951-016-0183-z PMC4839071 27102110

[CIT0013] Franco MS, Roque MC, de Barros ALB, et al. (2019). Investigation of the antitumor activity and toxicity of long-circulating and fusogenic liposomes co-encapsulating paclitaxel and doxorubicin in a murine breast cancer animal model. Biomed Pharmacother 109:1728–39. 30551427 10.1016/j.biopha.2018.11.011

[CIT0014] Gawde KA, Sau S, Tatiparti K, et al. (2018). Paclitaxel and di-fluorinated curcumin loaded in albumin nanoparticles for targeted synergistic combination therapy of ovarian and cervical cancers. Colloids Surf B Biointerfaces 167:8–19. 29625422 10.1016/j.colsurfb.2018.03.046

[CIT0015] Gohler S, Da Silva Filho MI, Johansson R, et al. (2017). Functional germline variants in driver genes of breast cancer. Cancer Causes Control 28:259–71. 28238063 10.1007/s10552-017-0849-3

[CIT0016] Hazeldine J, Harris P, Chapple IL, et al. (2014). Impaired neutrophil extracellular trap formation: a novel defect in the innate immune system of aged individuals. Aging Cell 13:690–8. 24779584 10.1111/acel.12222 PMC4326942

[CIT0017] Hu J, Yuan XW, Wang F, et al. (2021). The progress and perspective of strategies to improve tumor penetration of nanomedicines. Chin Chem Lett 32:1341–7.

[CIT0018] Jin X, Zhang J, Jin X, et al. (2020). Folate receptor targeting and cathepsin B-sensitive drug delivery system for selective cancer cell death and imaging. ACS Med Chem Lett 11:1514–20. 32832017 10.1021/acsmedchemlett.0c00031 PMC7430950

[CIT0019] Khalifa AM, Elsheikh MA, Khalifa AM, et al. (2019). Current strategies for different paclitaxel-loaded nano-delivery systems towards therapeutic applications for ovarian carcinoma: a review article. J Control Release 311–312:125–37. 10.1016/j.jconrel.2019.08.034 31476342

[CIT0020] Kumari P, Ghosh B, Biswas S. (2016). Nanocarriers for cancer-targeted drug delivery. J Drug Target 24:179–91. 26061298 10.3109/1061186X.2015.1051049

[CIT0021] Levy-Nissenbaum E, Radovic-Moreno AF, Wang AZ, et al. (2008). Nanotechnology and aptamers: applications in drug delivery. Trends Biotechnol 26:442–9. 18571753 10.1016/j.tibtech.2008.04.006

[CIT0022] Ma Y, Wang Y, Song B. (2019). Griffipavixanthone induces apoptosis of human breast cancer MCF-7 cells *in vitro*. Breast Cancer 26:190–7. 30259331 10.1007/s12282-018-0912-2

[CIT0023] Maheo K, Vibet S, Steghens JP, et al. (2005). Differential sensitization of cancer cells to doxorubicin by DHA: a role for lipoperoxidation. Free Radic Biol Med 39:742–51. 16109304 10.1016/j.freeradbiomed.2005.04.023

[CIT0024] Mohammad RM, Muqbil I, Lowe L, et al. (2015). Broad targeting of resistance to apoptosis in cancer. Semin Cancer Biol 35:S78–S103. 25936818 10.1016/j.semcancer.2015.03.001 PMC4720504

[CIT0025] Monteiro CAP, Oliveira A, Silva RC, et al. (2020). Evaluating internalization and recycling of folate receptors in breast cancer cells using quantum dots. J Photochem Photobiol B 209:111918. 32531690 10.1016/j.jphotobiol.2020.111918

[CIT0026] Mouradian M, Kikawa KD, Dranka BP, et al. (2015). Docosahexaenoic acid attenuates breast cancer cell metabolism and the Warburg phenotype by targeting bioenergetic function. Mol Carcinog 54:810–20. 24729481 10.1002/mc.22151

[CIT0027] Muhamad N, Plengsuriyakarn T, Na-Bangchang K. (2018). Application of active targeting nanoparticle delivery system for chemotherapeutic drugs and traditional/herbal medicines in cancer therapy: a systematic review. Int J Nanomedicine 13:3921–35. 30013345 10.2147/IJN.S165210 PMC6038858

[CIT0028] Mussi SV, Sawant R, Perche F, et al. (2014). Novel nanostructured lipid carrier co-loaded with doxorubicin and docosahexaenoic acid demonstrates enhanced *in vitro* activity and overcomes drug resistance in MCF-7/Adr cells. Pharm Res 31:1882–92. 24522814 10.1007/s11095-013-1290-2

[CIT0029] Mussi SV, Silva RC, Oliveira MC, et al. (2013). New approach to improve encapsulation and antitumor activity of doxorubicin loaded in solid lipid nanoparticles. Eur J Pharm Sci 48:282–90. 23178339 10.1016/j.ejps.2012.10.025

[CIT0030] Niu F, Yan J, Ma B, et al. (2018). Lanthanide-doped nanoparticles conjugated with an anti-CD33 antibody and a p53-activating peptide for acute myeloid leukemia therapy. Biomaterials 167:132–42. 29571049 10.1016/j.biomaterials.2018.03.025 PMC5889738

[CIT0031] Oroojalian F, Babaei M, Taghdisi SM, et al. (2018). Encapsulation of thermo-responsive gel in pH-sensitive polymersomes as dual-responsive smart carriers for controlled release of doxorubicin. J Control Release 288:45–61. 30171978 10.1016/j.jconrel.2018.08.039

[CIT0032] Park J, Choi Y, Chang H, et al. (2019). Alliance with EPR effect: combined strategies to improve the EPR effect in the tumor microenvironment. Theranostics 9:8073–90. 31754382 10.7150/thno.37198 PMC6857053

[CIT0033] Ponde NF, Zardavas D, Piccart M. (2019). Progress in adjuvant systemic therapy for breast cancer. Nat Rev Clin Oncol 16:27–44. 30206303 10.1038/s41571-018-0089-9

[CIT0034] Qin T, Xu X, Zhang Z, et al. (2020). Paclitaxel/sunitinib-loaded micelles promote an antitumor response *in vitro* through synergistic immunogenic cell death for triple-negative breast cancer. Nanotechnology 31:365101. 32434167 10.1088/1361-6528/ab94dc

[CIT0035] Ren X, Wang N, Zhou Y, et al. (2021). An injectable hydrogel using an immunomodulating gelator for amplified tumor immunotherapy by blocking the arginase pathway. Acta Biomater 124:179–90. 33524560 10.1016/j.actbio.2021.01.041

[CIT0036] Rescigno T, Capasso A, Tecce MF. (2016). Effect of docosahexaenoic acid on cell cycle pathways in breast cell lines with different transformation degree. J Cell Physiol 231:1226–36. 26480024 10.1002/jcp.25217

[CIT0037] Serini S, Ottes Vasconcelos R, Nascimento Gomes R, et al. (2017). Protective effects of omega-3 PUFA in anthracycline-induced cardiotoxicity: a critical review. Int J Mol Sci 18:2689. 10.3390/ijms18122689 PMC5751291 29231904

[CIT0038] Shen M, Huang Y, Han L, et al. (2012). Multifunctional drug delivery system for targeting tumor and its acidic microenvironment. J Control Release 161:884–92. 22587941 10.1016/j.jconrel.2012.05.013

[CIT0039] Siddiqui RA, Harvey KA, Xu Z, et al. (2011). Docosahexaenoic acid: a natural powerful adjuvant that improves efficacy for anticancer treatment with no adverse effects. Biofactors 37:399–412. 22038684 10.1002/biof.181

[CIT0040] Siegel RL, Miller KD, Jemal A. (2020). Cancer statistics, 2020. CA Cancer J Clin 70:7–30. 31912902 10.3322/caac.21590

[CIT0041] Sun H, Su J, Meng Q, et al. (2016). Cancer-cell-biomimetic nanoparticles for targeted therapy of homotypic tumors. Adv Mater 28:9581–8. 27628433 10.1002/adma.201602173

[CIT0042] Sung H, Ferlay J, Siegel RL, et al. (2021). Global Cancer Statistics 2020: GLOBOCAN estimates of incidence and mortality worldwide for 36 cancers in 185 countries. CA Cancer J Clin 71:209–49. 33538338 10.3322/caac.21660

[CIT0043] Tang H, Chen J, Wang L, et al. (2020). Co-delivery of epirubicin and paclitaxel using an estrone-targeted PEGylated liposomal nanoparticle for breast cancer. Int J Pharm 573:118806. 31678519 10.1016/j.ijpharm.2019.118806

[CIT0044] Untch M, Jackisch C, Schneeweiss A, et al. (2016). Nab-paclitaxel versus solvent-based paclitaxel in neoadjuvant chemotherapy for early breast cancer (GeparSepto-GBG 69): a randomised, phase 3 trial. Lancet Oncol 17:345–56. 26869049 10.1016/S1470-2045(15)00542-2

[CIT0045] Wang J, Li D, Cang H, et al. (2019). Crosstalk between cancer and immune cells: role of tumor-associated macrophages in the tumor microenvironment. Cancer Med 8:4709–21. 31222971 10.1002/cam4.2327 PMC6712467

[CIT0046] Wang TM, Chen CJ, Lee TS, et al. (2011). Docosahexaenoic acid attenuates VCAM-1 expression and NF-κB activation in TNF-α-treated human aortic endothelial cells. J Nutr Biochem 22:187–94. 20573493 10.1016/j.jnutbio.2010.01.007

[CIT0047] Wong RS. (2011). Apoptosis in cancer: from pathogenesis to treatment. J Exp Clin Cancer Res 30:87. 21943236 10.1186/1756-9966-30-87 PMC3197541

[CIT0048] Wu Q, Yang Z, Nie Y, et al. (2014). Multi-drug resistance in cancer chemotherapeutics: mechanisms and lab approaches. Cancer Lett 347:159–66. 24657660 10.1016/j.canlet.2014.03.013

[CIT0049] Yao Q, Gutierrez DC, Hoang NH, et al. (2017). Efficient codelivery of paclitaxel and curcumin by novel bottlebrush copolymer-based micelles. Mol Pharm 14:2378–89. 28605595 10.1021/acs.molpharmaceut.7b00278

[CIT0050] Yu W, Hu C, Gao H. (2021). Advances of nanomedicines in breast cancer metastasis treatment targeting different metastatic stages. Adv Drug Deliv Rev 178:113909. 34352354 10.1016/j.addr.2021.113909

[CIT0051] Yuan H, Miao J, Du YZ, et al. (2008). Cellular uptake of solid lipid nanoparticles and cytotoxicity of encapsulated paclitaxel in A549 cancer cells. Int J Pharm 348:137–45. 17714896 10.1016/j.ijpharm.2007.07.012

[CIT0052] Zhang X, Sun X, Li J, et al. (2011). Lipid nanoemulsions loaded with doxorubicin–oleic acid ionic complex: characterization, *in vitro* and *in vivo* studies. Pharmazie 66:496–505. 21812324

[CIT0053] Zheng X, Xie JZ, Zhang X, et al. (2021). An overview of polymeric nanomicelles in clinical trials and on the market. Chin Chem Lett 32:243–57.

