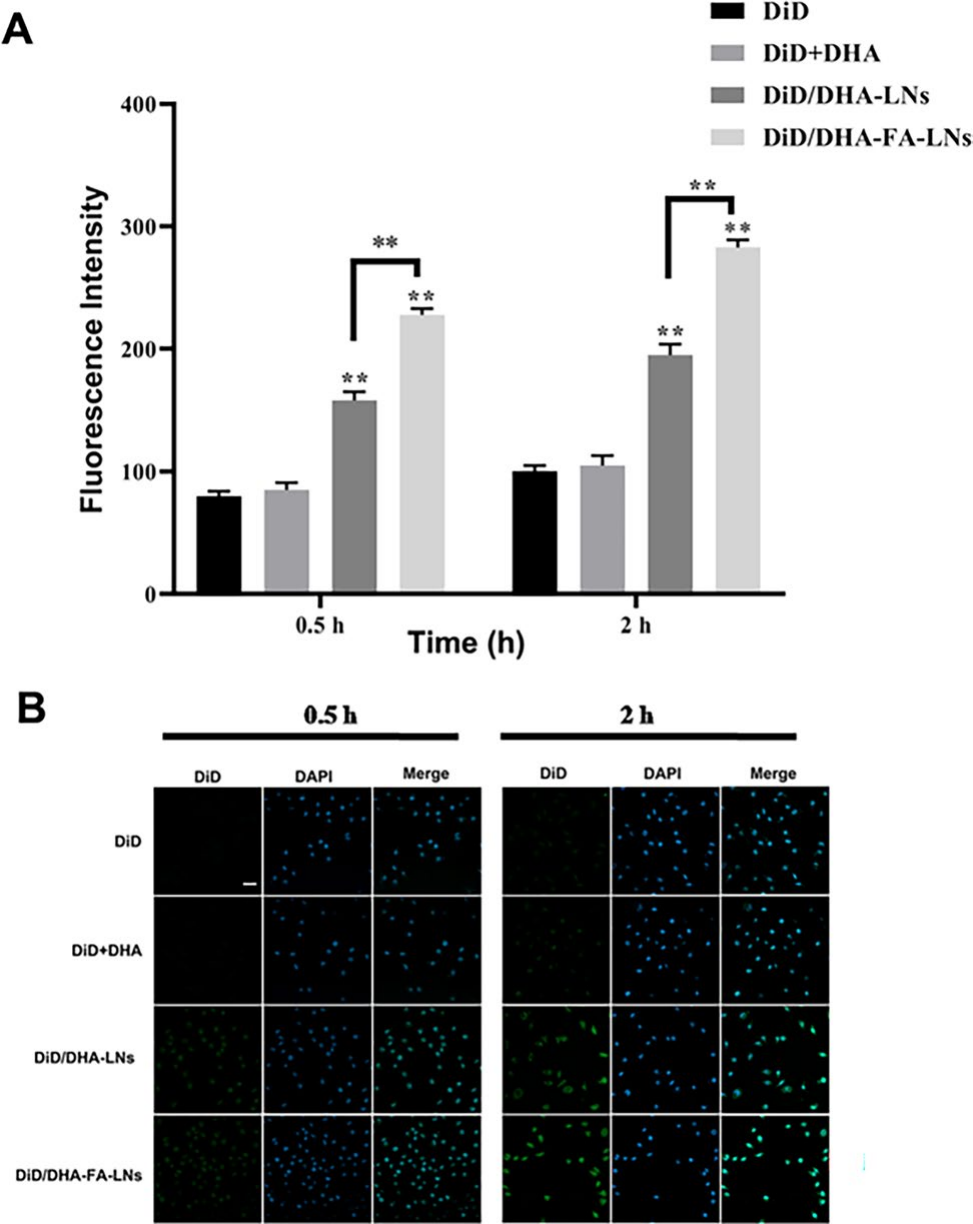# Correction notice

**DOI:** 10.1080/10717544.2024.2339792

**Published:** 2024-06-18

**Article title:** Co-delivery of paclitaxel (PTX) and docosahexaenoic acid (DHA) by targeting lipid nanoemulsions for cancer therapy

**Authors:** Bo Li, Tingfei Tan, Weiwei Chu, Ying Zhang, Yuanzi Ye, Shanshan Wang, Yan Qin, Jihui Tang & Xi Cao

**Journal:** *Drug Delivery*

**Bibliometrics:** Volume 29, Number 01, pages 75-88

**DOI:** https://doi.org/10.1080/10717544.2021.2018523

When this article was first published online, incorrect Figure 6 was published. This figure has since been replaced with the correct one, and the updated version of the article has been republished online. The corrected Figure 6 is displayed below:

**Figure:**