# Supplementary material for: Co-delivery of paclitaxel (PTX) and docosahexaenoic acid (DHA) by targeting lipid nanoemulsions for cancer therapy
Source: Drug Deliv. 2021 Dec 29;29(1):75–88. doi: 10.1080/10717544.2021.2018523 (PMC8735879; doi:10.1080/10717544.2021.2018523)
Supplement: Supplemental Material [file IDRD_A_2018523_SM4363.docx]

**Electronic Supporting Information**

**Co-delivery of paclitaxel (PTX) and docosahexaenoic acid (DHA) by targeting lipid nanoemulsions for cancer therapy**

Bo Li^1, 2*^, Tingfei Tan^1, 2*^, Weiwei Chu^1, 2^，Ying Zhang^3^， Yuanzi Ye^3^，Shanshan Wang^1, 2^，Yan Qin^4^，Jihui Tang^4*^，Xi Cao^1, 2*^

## Drug release of PTX-LNs and PTX-FA-LNs in vitro. The release profiles of PTX from PTX-FA-LNs were studied using a dialysis method, and PTX solution was prepared as control. At predetermined time intervals, 3 mL of aliquot from the external medium was taken out and replenished with an equal volume of fresh medium. The concentrations of PTX at each time point were determined as previously described, and the cumulative drug release percentage was calculated accordingly. The release kinetics of PTX-FA-LNs displayed a dual phase profile for PTX with more than 50% drugs released by 12 h. From 12 to 80 h, PTX was shown to release from LNs in a prolonged release manner (Figure S1).


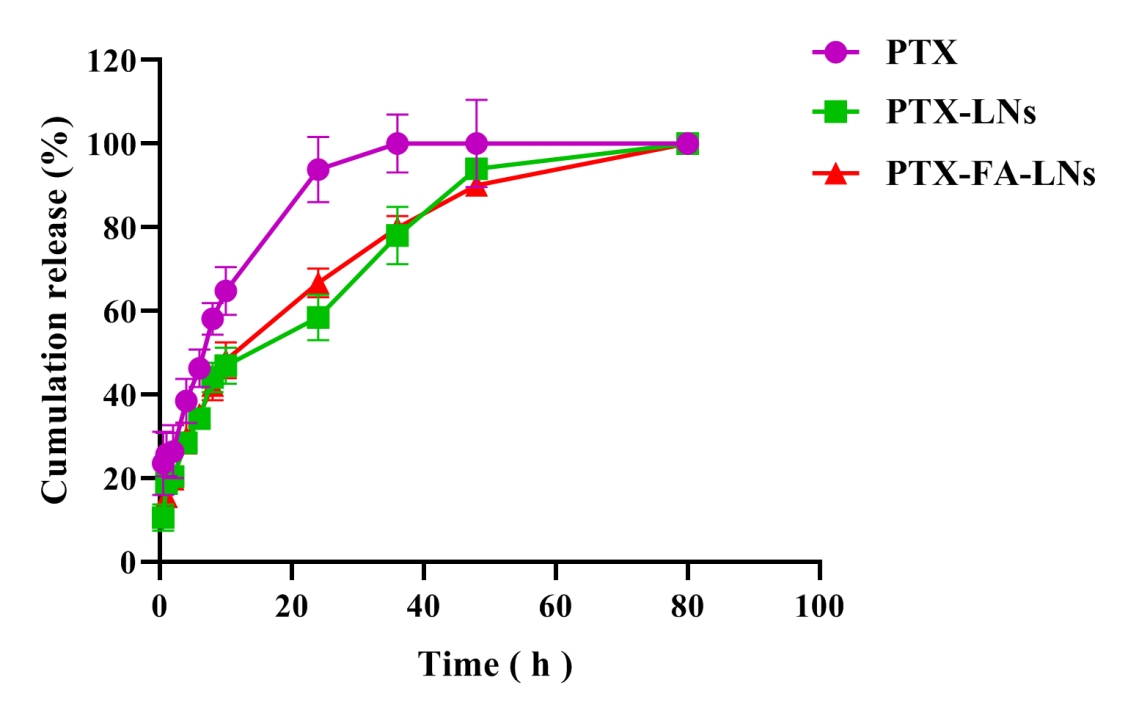


**Figure S1.** In vitro release profiles of PTX from PTX-LNs solution and PTX-FA-LNs. Data represent mean ± SD (n = 3).

## Cytotoxicity of PTX-LNs and PTX-FA-LNs. The cytotoxicity of PTX and FA, encapsulated in LNs, was investigated using MCF-7 breast cancer cells. At each concentration under investigation, PTX-FA-LNs and PTX-LNs showed much higher inhibitory effect compared to PTX solution in MCF-7 cells after 24 hrs of treatment (Figure S2). As demonstrated in Figure S2, when PTX and FA were combined with LNs (at the same concentrations above), a slight reduction in cell viabity was observed compared to PTX-LNs.


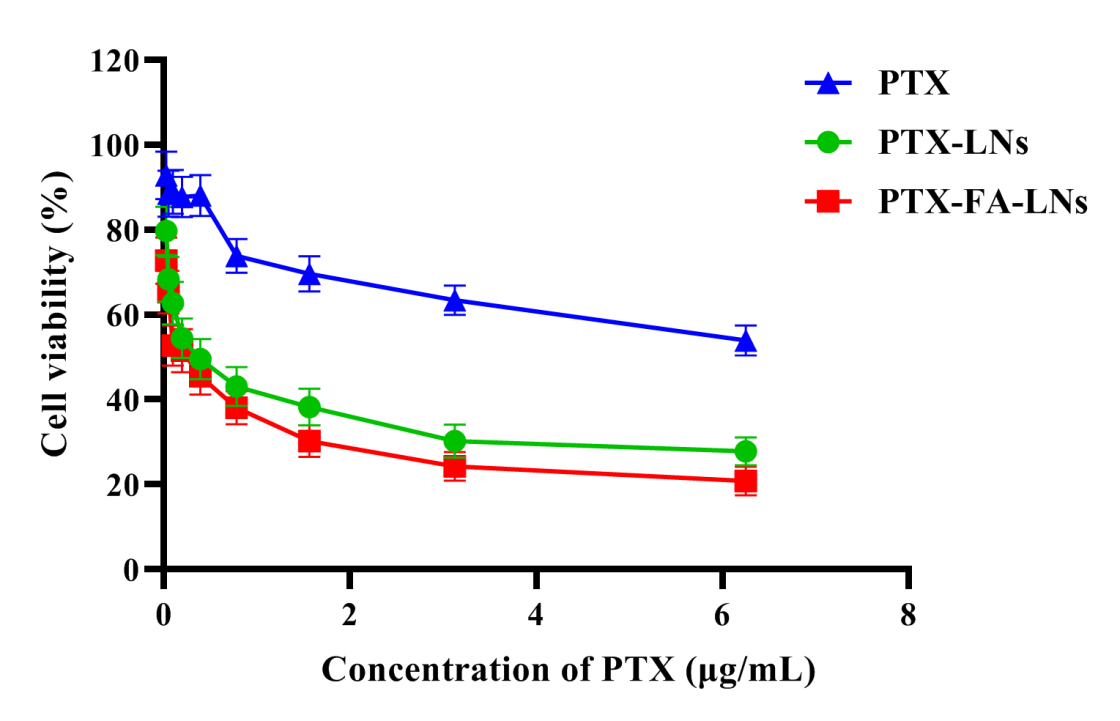


**Figure S2.** *In vitro* cell viability of PTX, PTX-LNs and PTX-FA-LNs in MCF-7 cells after 24 h of incubation.

## Cell apoptosis of PTX-FA-LNs. The administration of PTX-FA-LNs showed significantly higher apoptosis rate (69.23% ± 4.70%) in cells as compared to the treatments of PTX-LNs (55.95% ± 2.80%, p < 0.01), and PTX free group (40.54% ± 3.50%, p < 0.01). The results(Figure S3) revealed that PTX-FA-LNs induced significant cytotoxicity toward MCF-7 cells through apoptosis pathway. Thus, the lipid nanocarriers were more effective in reducing the capacity of the cells to divide to produce progeny after treatment, probably causing damage in chromosomes and interfering in cell death.


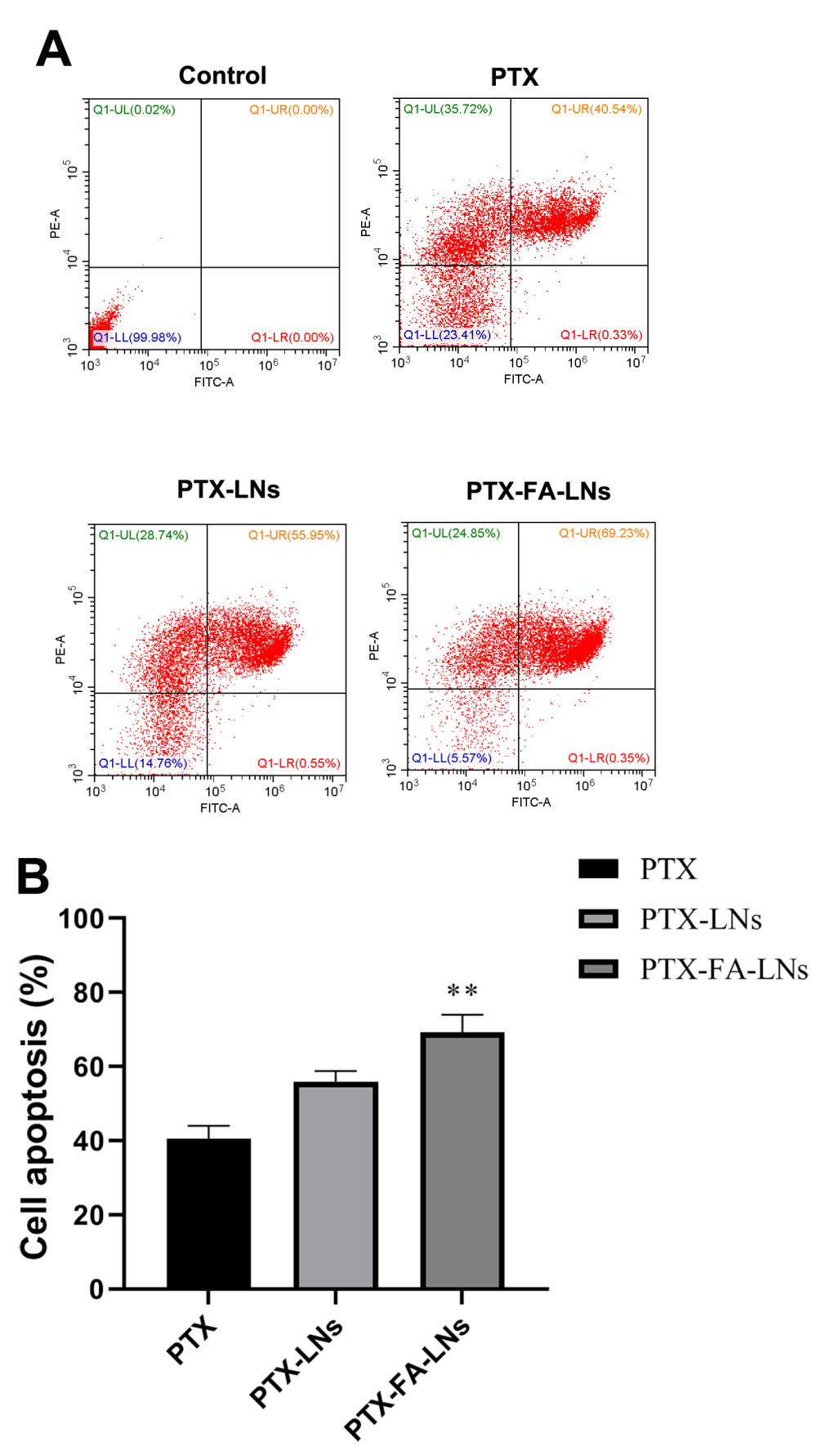


**Figure S3.** Cell apoptosis and necrosis were analyzed by flow cytometry using annexin V-FITC in combination with PI in MCF-7 cells. The quantification of apoptotic and necrotic cell percentages after treatment with different formulations in MCF-7 cells (***p* < 0.01).
